# Exposure of bighorn sheep to domestic goats colonized with *Mycoplasma ovipneumoniae* induces sub-lethal pneumonia

**DOI:** 10.1371/journal.pone.0178707

**Published:** 2017-06-07

**Authors:** Thomas E. Besser, E. Frances Cassirer, Kathleen A. Potter, William J. Foreyt

**Affiliations:** 1 Department of Veterinary Microbiology and Pathology, Washington State University College of Veterinary Medicine, Pullman, WA, United States of America; 2 Idaho Department of Fish and Game, Lewiston, ID, United States of America; Université de Sherbrooke, CANADA

## Abstract

**Background:**

Bronchopneumonia is a population limiting disease of bighorn sheep (*Ovis canadensis*) that has been associated with contact with domestic Caprinae. The disease is polymicrobial but is initiated by *Mycoplasma ovipneumoniae*, which is commonly carried by both domestic sheep (*O*. *aries*) and goats (*Capra aegagrus hircus*). However, while previous bighorn sheep comingling studies with domestic sheep have resulted in nearly 100% pneumonia mortality, only sporadic occurrence of fatal pneumonia was reported from previous comingling studies with domestic goats. Here, we evaluated the ability of domestic goats of defined *M*. *ovipneumoniae* carriage status to induce pneumonia in comingled bighorn sheep.

**Methodology/Principal findings:**

In experiment 1, three bighorn sheep naïve to *M*. *ovipneumoniae* developed non-fatal respiratory disease (coughing, nasal discharge) following comingling with three naturally *M*. *ovipneumoniae*-colonized domestic goats. Gross and histological lesions of pneumonia, limited to small areas on the ventral and lateral edges of the anterior and middle lung lobes, were observed at necropsies conducted at the end of the experiment. A control group of three bighorn sheep from the same source housed in isolation during experiment 1 remained free of observed respiratory disease. In experiment 2, three bighorn sheep remained free of observed respiratory disease while comingled with three *M*. *ovipneumoniae-*free domestic goats. In experiment 3, introduction of a domestic goat-origin strain of *M*. *ovipneumoniae* to the same comingled goats and bighorn sheep used in experiment 2 resulted in clinical signs of respiratory disease (coughing, nasal discharge) in both host species. At the end of experiment 3, gross and histological evidence of pneumonia similar to that observed in experiment 1 bighorn sheep was observed in both affected bighorn sheep and domestic goats.

**Conclusions/Significance:**

*M*. *ovipneumoniae* strains carried by domestic goats were transmitted to comingled bighorn sheep, triggering development of pneumonia. However, the severity of the disease was markedly milder than that seen in similar experiments with domestic sheep strains of the bacterium.

## Introduction

Historical and contemporary field observations suggest that contacts with domestic sheep are followed by pneumonia outbreaks in previously healthy bighorn sheep populations [1–3]. Supporting these observations, >95% of 90 bighorn sheep in eleven studies involving contact with domestic sheep suffered fatal pneumonia within 100 days, as reviewed in [4]. However, the potential role of domestic goats in transmitting pneumonia pathogens to bighorn sheep is less clear, with relatively fewer observed bighorn sheep pneumonia outbreaks following domestic goat contacts [5–7] and with relatively little fatal bighorn sheep pneumonia observed in two experimental comingling studies with domestic goats (22% of 9 bighorn sheep) [5, 8]. Domestic goats may transmit respiratory pathogens when they contact bighorn sheep on or near bighorn sheep home ranges as stray animals from farmsteads, or when used for weed control, or when commmercial operations are grazed on public lands in or near bighorn sheep home ranges, or when used as pack animals supporting back-country recreation. In addition, bighorn sheep may encounter domestic goats on private lands within or adjacent to bighorn ranges. Therefore, it is important to understand the risk that contact with domestic goats may pose for bighorn sheep health.

*Mycoplasma ovipneumoniae*, proposed as the primary pathogen that triggers bighorn sheep pneumonia outbreaks [9, 10] followed by persistently impaired recruitment resulting from recurrent lamb pneumonia [11, 12], has a host range limited to Caprinae [13]. Distinct host-specific clades of *M*. *ovipneumoniae* have recently been reported in domestic sheep and goats [7, 14]. In a Washington state survey of goat farms adjacent to bighorn sheep habitat, this pathogen was carried asymptomatically by animals on 7 of 16 goat farms, and by 58% of individual goats on positive farms [15].

Like bighorn sheep pneumonia, respiratory disease of domestic sheep and goats is polymicrobial, frequently including *M*. *ovipneumoniae* (and perhaps also *Mycoplasma arginini*), *Mannheimia haemolytica*, *Pasteurella multocida*, and other pathogens. Chronic and persistent coughing is the main clinical sign observed associated with *M*. *ovipneumoniae* infection of domestic lambs, which is often described as summer pneumonia in Australia and New Zealand, or as coughing syndrome in the United States [13, 16]. Clinical severity ranges from mild illness associated with reduced growth rates to fatal polymicrobial pneumonia [13, 17]. *M*. *ovipneumoniae* has also been associated with outbreaks of severe respiratory disease in goats [13, 18, 19].

The *M*. *ovipneumoniae* carriage status of the animals used in previously published experimental domestic goat-bighorn sheep comingling studies was not defined [5, 8] and so it is possible that the low death rate reported in those studies was due to the absence of *M*. *ovipneumoniae* in the comingled goats. Here we tested the hypothesis that exposure to domestic goats carrying *M*. *ovipneumoniae* results in epizootic pneumonia in bighorn sheep by conducting a series of experimental comingling studies with domestic goats of defined *M*. *ovipneumoniae* carriage status.

## Materials and methods

### Ethics statement

This study was carried out in accordance with the recommendations in the Guide for the Care and Use of Laboratory Animals of the National Institutes of Health and in conformance with United States Department of Agriculture animal research guidelines, under protocols #03793 and #04482 approved by the Washington State University Institutional Animal Care and Use Committee. As described in those protocols, euthanasia was to be performed by intravenous injection of sodium pentobarbital for animals observed to be in severe distress associated with pneumonia during the study and prior to any necropsy examination for surviving animals at the end of each experiment.

### Experimental animals

Six healthy, *M*. *ovipneumoniae-*unexposed, pregnant adult bighorn ewes were provided by the Washington Department of Fish and Wildlife on January 25, 2014 from the Clemans Mountain population for these studies. This population had previously been confirmed to be uninfected and unexposed to *M*. *ovipneumoniae* as determined by repeated testing with both competitive enzyme-linked immunosorbent assay (cELISA) serology and nasal swab polymerase chain reaction (PCR) testing [20]. Blood and nasal swab samples collected from the six experimental animals on the day of capture were also negative by *M*. *ovipneumoniae* cELISA and PCR tests.The ewes were randomly allocated to two experimental groups of three animals each. Sampling or moving the bighorn sheep was conducted under anesthesia (butorphanol tartrate, azaperone tartrate, and medetomidine hydrochloride, reversed with atipamezole and naltrexone, BAM kit, Wildlife Pharmaceuticals, Inc. Windsor CO). Six domestic goats were purchased from three local private owners for use in the experiments. Domestic goats used in experiment 1 (group 1) included a 5+ year Saanen wether previously used as a pack animal, a 1 year old mixed breed wether intended for slaughter, and a 1 year old Boer wether from a herd used for weed and brush control. All were confirmed nasal carriers of *M*. *ovipneumoniae* based on PCR testing of nasal swab samples on at least two occasions prior to the study. Domestic goats used in experiments 2 and 3 (group 2) included one yearling Boer female and two yearling LaMancha wethers, all negative for *M*. *ovipneumoniae* by nasal swab PCR when purchased. Because they originated in a *M*. *ovipneumoniae-*positive herd, group 2 domestic goats were placed in isolation from other small ruminants and were serially re-tested for *M*. *ovipneumoniae* nasal carriage (5 times over a 4 week period) to confirm their negative status prior to the start of experiment 2.

### Experimental design

Three experiments were conducted in which domestic goats (DG) of known *M*. *ovipneumoniae* status were comingled with susceptible bighorn sheep (BHS). In each experiment, comingled animals shared feed and water sources (pelleted hay supplemented with a corn/oats/barley mixture, free choice trace mineral salt, and a single frost-protected water source in each pen). Single roofed shelters were available in each pen, and plywood sheets covered the pen fences at the corners to provide windbreaks. All animals were observed daily.

#### Experiment 1

BHS group 1 (BHS29, BHS32, and BHS33; *M*. *ovipneumoniae-*negative) was comingled in a 372 m^2^ pen (pen 1) with group 1 DG (DG1, DG2, and DG3; *M*. *ovipneumoniae-*positive) beginning February 18, 2014. During experiment 1, BHS group 2 (BHS27, BHS28, and BHS31; *M*. *ovipneumoniae-*negative) were housed in a separate 232 m^2^ pen (pen 2) isolated >30 m from any other domestic or wild sheep or goats. All animals were observed closely for signs of respiratory disease for a 100 day period. On May 30, 2014, BHS group 1 animals were euthanized and subjected to complete necropsy examinations. DG group 1, having shown no clinical signs of respiratory disease during the experiment, were donated to the Washington State University veterinary teaching program.

#### Experiment 2

After group 1 animals were removed at the end of experiment 1, pen 1 was left empty for 40 days before BHS group 2 (*M*. *ovipneumoniae-*negative) was added on July 7, 2014. BHS group 2 composition was modified by the loss of BHS27 and by the addition of BHS28L (a lamb born to BHS28 on April 18, 2014 during the first experiment. On September 10, 2014, 65 days after BHS group 2 were placed in pen 1, DG group 2 (DG4, DG5, and DG6; *M*. *ovipneumoniae-*negative) was comingled to begin experiment 2, in which the comingled animals were again closely observed for signs of respiratory disease for a 100 day period.

#### Experiment 3

In experiment 3, a strain of *M*. *ovipneumoniae* obtained by nasal wash from a domestic goat (originating on a different farm than the source farms of DG groups 1 and 2) was introduced into the comingled BHS and DG from experiment 2, as follows: DG4 and DG5 were removed to an isolation pen where they were each inoculated with *M*. *ovipneumoniae* by instillation of 10 ml of ceftiofur-treated nasal wash fluids into conjunctivae and nasal passages. After confirmation of colonization by positive nasal swab PCR tests on days 3 and 7 following inoculation, they were re-commingled in pen 1 with *M*. *ovipneumoniae-*negative DG6 and BHS group 2 on February 16, 2015. The commingled animals were observed closely for signs of respiratory disease for a 100 day period. At the end of experiment 3, both BHS goup 2 and DG group 2 were euthanized and subjected to complete necropsy examinations, as all had demonstrated signs of respiratory disease during the experiment.

### Biosecurity

The study pens used for these experiments had not housed any BHS, DG or domestic sheep for >12 months prior to initiation of these experiments. Routine biosecurity measures during the experiments included broad physical separation (>1 km) and entirely separate animal care staff for pens 1 and 2. Animal care staff and researchers utilized personal clean protective equipment (coveralls, boots, and gloves) when entering study pens. Research staff occasionally entered both pens 1 and 2 on the same day, but if so always entered the *M*. *ovipneumoniae-*free pen 2 first, and changed personal protective equipment before entering pen 1.

### Clinical scores

Clinical signs of respiratory disease were scored appproximately 3 times per week using a point system: anorexia (1 point), nasal discharge (1 point), cough (2 points), nose licking (1 point), head shaking (1 point), ear paresis (1 point) and weakness/incoordination (1 point), as we have done previously [21].

### Microbiological testing

Routine microbiological testing performed by the Washington Animal Diagnostic Laboratory (WADDL, accredited by the American Association of Veterinary Laboratory Diagnosticians), included detection of *M*. *ovipneumoniae*-specific antibodies in serum samples using competitive enzyme-linked immunosorbent assays (cELISA) [9, 22], detection of *M*. *ovipneumoniae*-specific DNA sequences by polymerase chain reaction (PCR) testing of nasal or bronchial swab eluates, culture-enriched swab samples (mycoplasma broth, 72 hrs, 35 C), or lung tissue samples [23, 24], detection of Pasteurellaceae in pharyngeal swab samples by conventional aerobic bacteriologic cultures and MALDI-TOF (Biotyper, Bruker, Woodlands TX) [25], and detection of neutralizing antibodies in serum samples indicating exposure to parainfluenza-3 and respiratory syncytial viruses by virus neutralization assays. PCR tests specific for detection of *M*. *haemolytica*, *B*. *trehalosi*, and *P*. *multocida*, and *lktA* (the gene encoding the principal virulence factor of *M*. *haemolytica* and *B*. *trehalosi*) were applied to DNA extracted from pneumonic lung tissues using previously described methods [26]. The 16S–23S ribosomal operon intergenic spacer (IGS) regions of *M*. *ovipneumoniae* recovered from animals in these studies were PCR amplified and their DNA sequences determined in order to track transmission of specific *M*. *ovipneumoniae* strains (GenBank accession numbers pending) [7, 27].

## Results

### Experiment 1

**Fig 1:**
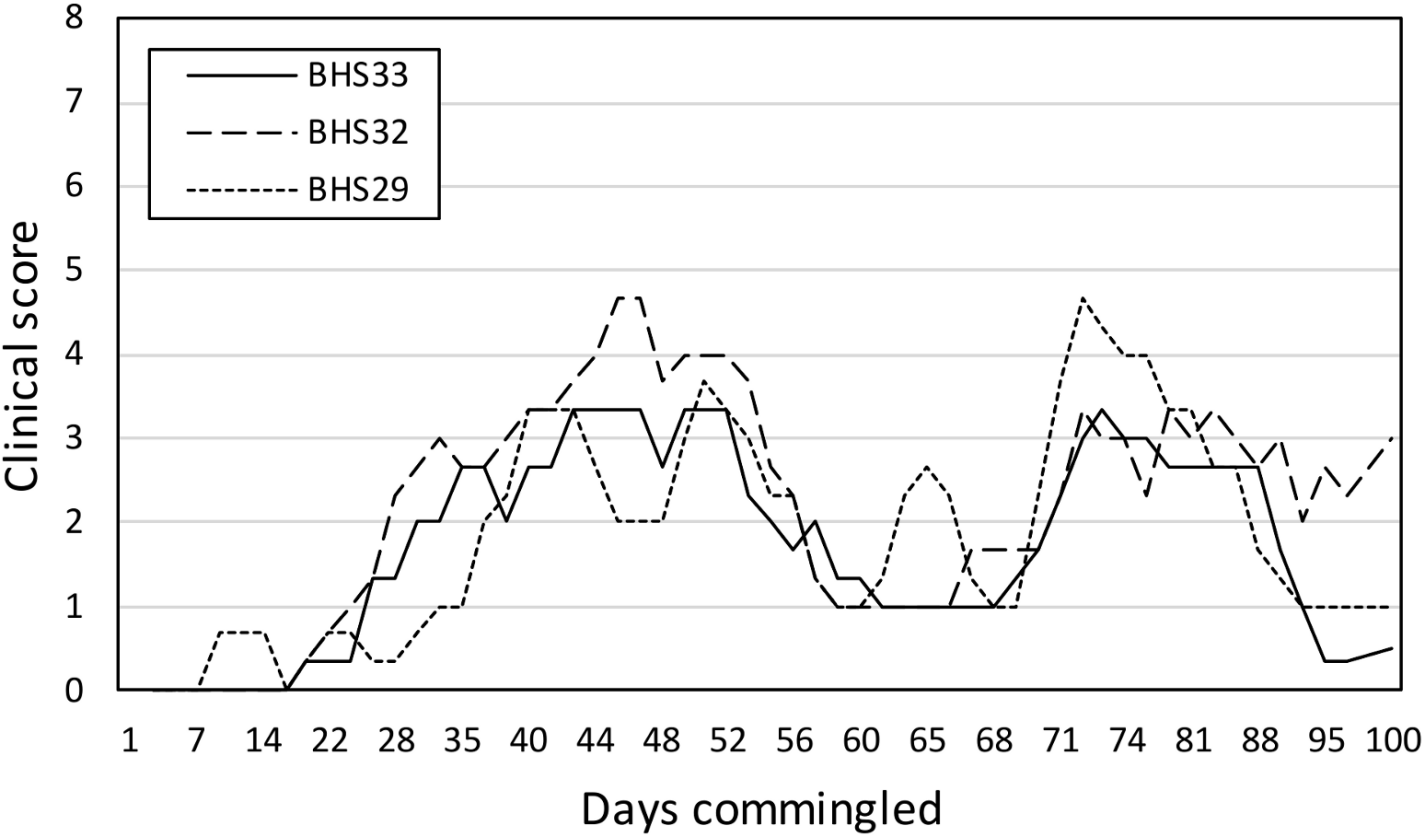
Clinical scores exhibited by bighorn sheep commingled with *M*. *ovipneumoniae* carrier domestic goats in Experiment 1. Three-day rolling average summed clinical scores based on observed anorexia, nasal discharge, cough, dyspnea, head shaking, ear paresis and weakness/incoordination. *M*. *ovipneumoniae* colonized DG were comingled with naïve BHS on day 0.

All animals appeared clinically normal at the start of experiment 1. All BHS were negative for carriage (PCR testing of nasal swabs) and exposure to *M*. *ovipneumoniae* (cELISA testing of serum samples) (Table 1). DG were confirmed as carriers of *M*. *ovipneumoniae* based on detection of the bacterium by PCR on repeated nasal swab samples. Diverse pharyngeal Pasteurellaceae were detected in both BHS and DG during experiment 1 (Table 1). Group 1 BHS (BHS29, BHS32, and BHS33) and group 1 DG (DG1, DG2, and DG3) were commingled in pen 1 to begin the experiment, while group 2 BHS (BHS27, BHS28, and BHS31) were maintained in isolation in pen 2. Beginning 2–3 weeks after commingling, all group 1 BHS began showing signs of respiratory disease, including increased nasal discharge and coughing (Fig 1, S1 Table). Clinical signs of middle ear involvement (head shaking, ear droop) were rarely seen. After 70 days of illness, group 1 BHS exhibited decreasing clinical signs of respiratory tract disease, and group 1 DG remained clinically normal throughout the experiment. Group 1 BHS were euthanized and necropsied after 100 days of commingling. Uncommingled group 2 BHS remained healthy in pen 2 throughout experiment 1. At the end of experiment 1, the *M*. *ovipneumoniae-*negative status of group 2 BHS was re-confirmed by PCR and cELISA serology. BHS27 subsequently died from aspiration pneumonia, the onset of which followed the anesthesia conducted to permit sampling after experiment 1; at necropsy, BHS27 was also PCR- and ELISA-negative for *M*. *ovipneumoniae*.

**Table 1:** Microbiological status and pathologic lesions of animals in experiment 1.

Animal	Pen	Stage	Gross necropsy^1^	*M*. *ovipneumoniae*^2^		*Pasteurellaceae (lktA)*^3^
				PCR	cELISA (%I)	
BHS29	1	Pre	-	NotDet	NotDet (-12%)	P: Btre, Mhae+
		Post	LC (15% r; 5% l), CSE	Det: B, CS	Det (75%)	L: Pmul
BHS32	1	Pre	-	NotDet	NotDet (-13%)	P: Btre+
		Post	LC (5% l), CSE	Det: N, L	Det (60%)	L: NotDet
BHS33	1	Pre	-	NotDet	NotDet (3%)	P: Mhae+
		Post	LC (1% r) PA ® CSE	Det: B, N	Det (82%)	L: Pspp
DG1	1	Both	-	Det: N	-	P: Btre, Mhae+
DG2	1	Both	-	Det: N	-	P: Mhae+
DG3	1	Both	-	Det: N	-	P: Btre, Mhae+
BHS27	2	Both	-	NotDet	NotDet (-7–3%)	P: Btre, Mhae, Psp
BHS28	2	Both	-	NotDet	NotDet (13–26%)	P: Btre, Mhae+, Pmul, Psp
BHS31	2	Both	-	NotDet	NotDet (7–16%)	P: Btre, Psp

^1^ - = not done; LC = lung consolidation; CSE = cornual sinus exudate; PA = pleural adhesions; l = left lung; r = right lung.

^2^%I = Percent inhibition; NotDet = not detected; Det = detected; N = nasal passage swab; B = bronchus swab; CS = cornual sinus swab; L = lung tissue;— = not done.

^3^ Detection of Pasteurellaceae by either bacteriologic culture or PCR; P = pharyngeal swab; L = lung tissue; Btre = *Bibersteinia trehalosi;* Mhae = *Mannheimia haemolytica;* Pmul = *Pasteurella multocida;* Psp = *Pasteurella* species; + = *lktA* PCR positive; NotDet = No Pasteurellaceae detected.

All BHS ewes lambed during experiment 1. All lambs born to group 1 (goat-commingled, *M*. *ovipneumoniae-*positive) BHS died at less than 7 days of age, with signs of ocular, systemic and/or gastrointestinal tract disease (BHS29L, BHS32L, and BHS33L; Table 2). At necropsy, none of these lambs had detectable pneumonia either grossly or histologically. The twin lambs born to group 2 (non-commingled; *M*. *ovipneumoniae-*negative) ewe BHS31 (BHS31L1 and BHS31L2) also died at 4 and 7 days of age, respectively, with signs of ocular, systemic and/or enteric disease. The lamb of group 2 BHS27 (BHS27L) was euthanized at 45 days of age necessitated by intractable diarrheal disease, while the lamb of BHS28 (BHS28L) survived.

**Table 2:** Outcomes and microbiological findings of lambs born during experiment 1.

Animal ID	Pen	Outcome^1^	*M*. *ovipneumoniae*^2^		*Pasteurellaceae* (*lktA*)^3^
			PCR	cELISA (% I)	
BHS29L	1	C (d6), death (d7)	Det: L N C	NotDet (0%)	P, L: Btre; C: Mhae+
BHS32L	1	C, D, death (d5)	Det: N	Det (50%)	P: Mhae+; C: Mhae+;
BHS33L	1	C, death (d7)	Det: N, B	Ind (49%)	P: Msp, Pmul; C: Mhae+; L: NotDet
BHS27L	2	D, euthanized d45)	NotDet	NotDet (2%)	NotDet
BHS28L	2	D (d54), survived	NotDet	NotDet (<4%)	P: Btre, Msp
BHS31L1	2	Death (d4)	NotDet	Not done	L: Mhae
BHS31L2	2	D, death (d10)	NotDet	NotDet (-9%)	L, C: Mhae+

^1^C = conjunctivitis, D = diarrhea.

^2^%I = Percent inhibition; Det = detected; NotDet = not detected; Ind = indeterminate result; L = lung tissue; N = nasal passage swab; C = Conjunctival swab; B = Bronchial swab

^3^ Detection of Pasteurellaceae by either bacteriologic culture or PCR; P = pharyngeal swab; L = lung tissue; C = conjunctival swab; Btre = *Bibersteinia trehalosi;* Mhae = *Mannheimia haemolytica;* Pmul = *Pasteurella multocida; M*sp = *Mannheimia* species; + = *lktA* PCR positive; NotDet = No Pasteurellaceae detected.

### Experiment 2

Group 2 DG, *M*. *ovipneumoniae-*free but carrying diverse pharyngeal Pasteurellaceae (Table 3), were commingled with group 2 BHS in pen #1. No respiratory disease signs were observed in either DG or BHS for >120 days during experiment 2.

**Table 3:** Microbiological status and pathologic lesions of animals in experiments 2 and 3.

Animal ID	Experiment	Gross necropsy^1^	*M*. *ovipneumoniae*^*2*^		*Pasteurellaceae* (*lktA*)^3^
			PCR^2^	cELISA (%I)	
BHS28	Pre 2	-	NotDet	NotDet (26%)	P: Btre, Mhae+, Psp
	Post 2 Pre 3	-	NotDet	NotDet (12%)	P: Btre, Msp, Psp
	Post 3	PA (r, l)	Det: N, B	Det (79%)	P: Btre; L: Btre, Pmul
BHS28L	Pre 2	-	NotDet	NotDet (4%)	P: Btre, Mhae+, Psp
	Post 2 Pre 3	-	NotDet	NotDet (15%)	P: Msp
	Post 3	LC (5% r, 1% l)	Det: N	Det (83%)	P: Btre; L: Btre
BHS31	Pre 2	-	NotDet	NotDet (7%)	P: Btre, Psp
	Post 2 Pre 3	-	NotDet	NotDet (16%)	P: NotDet
	Post 3	No lesions seen	Det: N, B	Det (77%)	P: Btre; L: Btre, Psp
DG4	Pre 2	-	NotDet (x5)	NotDet (29%)	P: Btre, Mhae+
	Post 2 Pre 3	-	NotDet	NotDet (20%)	P: Btre
	Post 3	LC (10% r, 5% l)	Det: N, B	Det (79%)	L: Mhae
DG5	Pre 2	-	NotDet (x5)	NotDet (21%)	P: Btre, Psp+
	Post 2 Pre 3	-	NotDet	NotDet (21%)	P: Mhae
	Post 3	LC (5% r, 5% l)	Det: N, B	Det (55%)	P: Btre; L: Mhae, Btre
DG6	Pre 2	-	NotDet (x5)	NotDet (28%)	P: Btre; Mhae+, Psp+
	Post 2 Pre 3	-	NotDet	NotDet (27%)	P: Btre, Mhae
	Post 3	No lesions seen	Det: N, B	Det (63%)	P: Btre; L: Btre, Mhae

^1^ - = not done, PA = pleural adhesions, LC = lung consolidation; l = left lung; r = right lung.

^**2**^%I = Percent inhibition; NotDet = not detected, Det = detected, N = nasal passage swab; B = bronchial swab.

^3^ Detection of Pasteurellaceae by either bacteriologic culture or PCR; P = pharyngeal swab; L = lung tissue; Btre = *Bibersteinia trehalosi;* Mhae = *Mannheimia haemolytica;* Pmul = *Pasteurella multocida; M*sp = *Mannheimia* species; Psp = *Pasteurella* species; + = *lktA* PCR positive; NotDet = No Pasteurellaceae detected by aerobic bacterial culture or by PCR.

### Experiment 3

**Fig 2:**
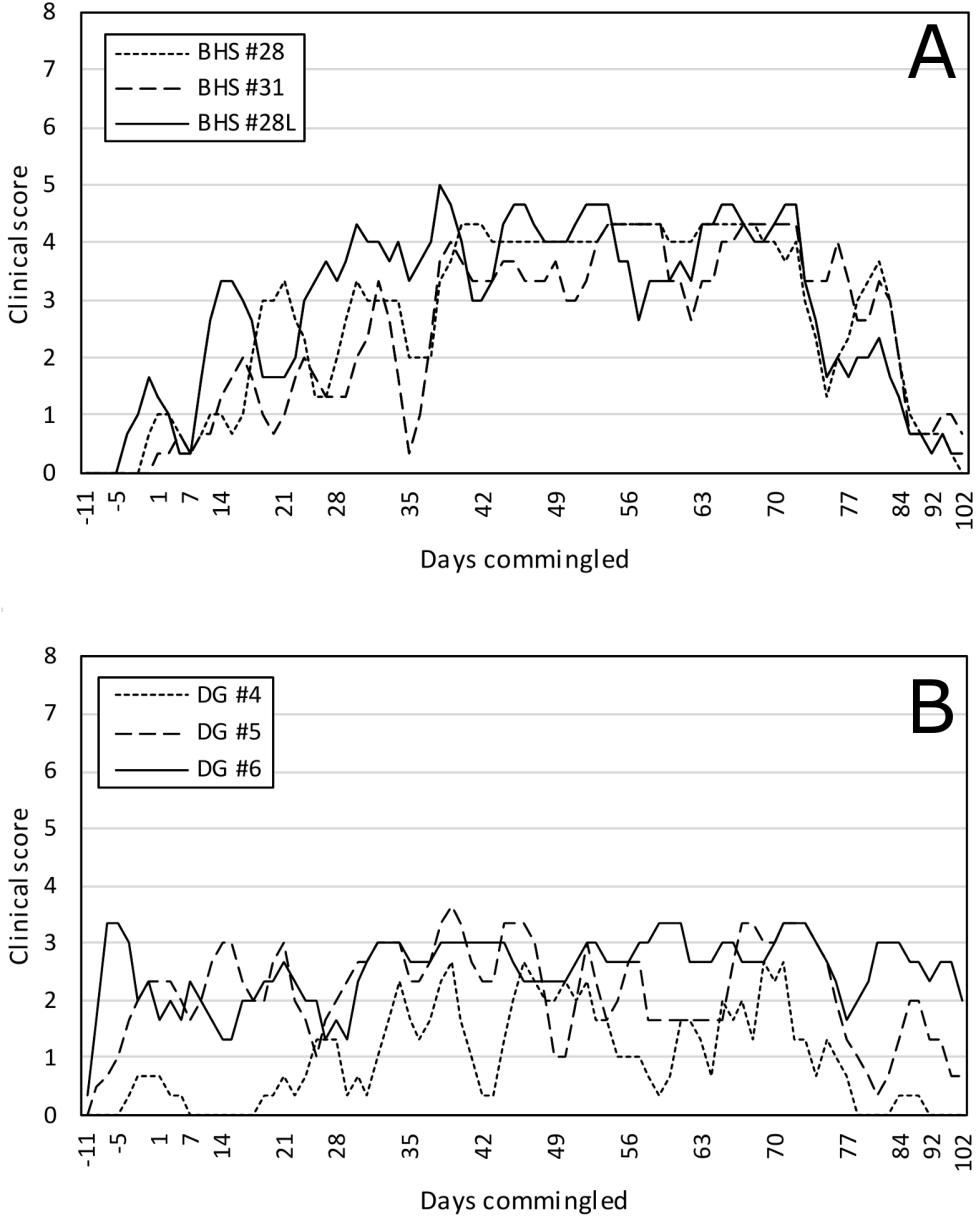
Clinical scores exhibited by commingled bighorn sheep and domestic goats in Experiment 3. Three-day rolling average summed clinical scores as described in Fig 1. (A) BHS; (B) DG. All BHS and DG in these experiments were naïve to *M*. *ovipneumoniae* prior to the experiment. DG4 and DG5 were exposed to *M*. *ovipneumoniae* on Day -11, and comingled with DG6 and BHS28, BHS28L and BHS31 on day 0.

Previously *M*. *ovipneumoniae-*free DG4 and DG5 began showing respiratory disease signs while still in isolation following *M*. *ovipneumoniae* inoculation and these signs continued after comingling in pen 1. All other pen 1 animals also developed signs of respiratory tract disease beginning shortly after re-comingling of DG4 and DG5 (Fig 2, S1 Table). The clinical respiratory disease scores of all animals in experiment 3 began diminishing after approximately 70 days of comingling. All animals were humanely euthanized for complete necropsy examinations at the end of experiment 3.

### Necropsy findings

**Fig 3:**
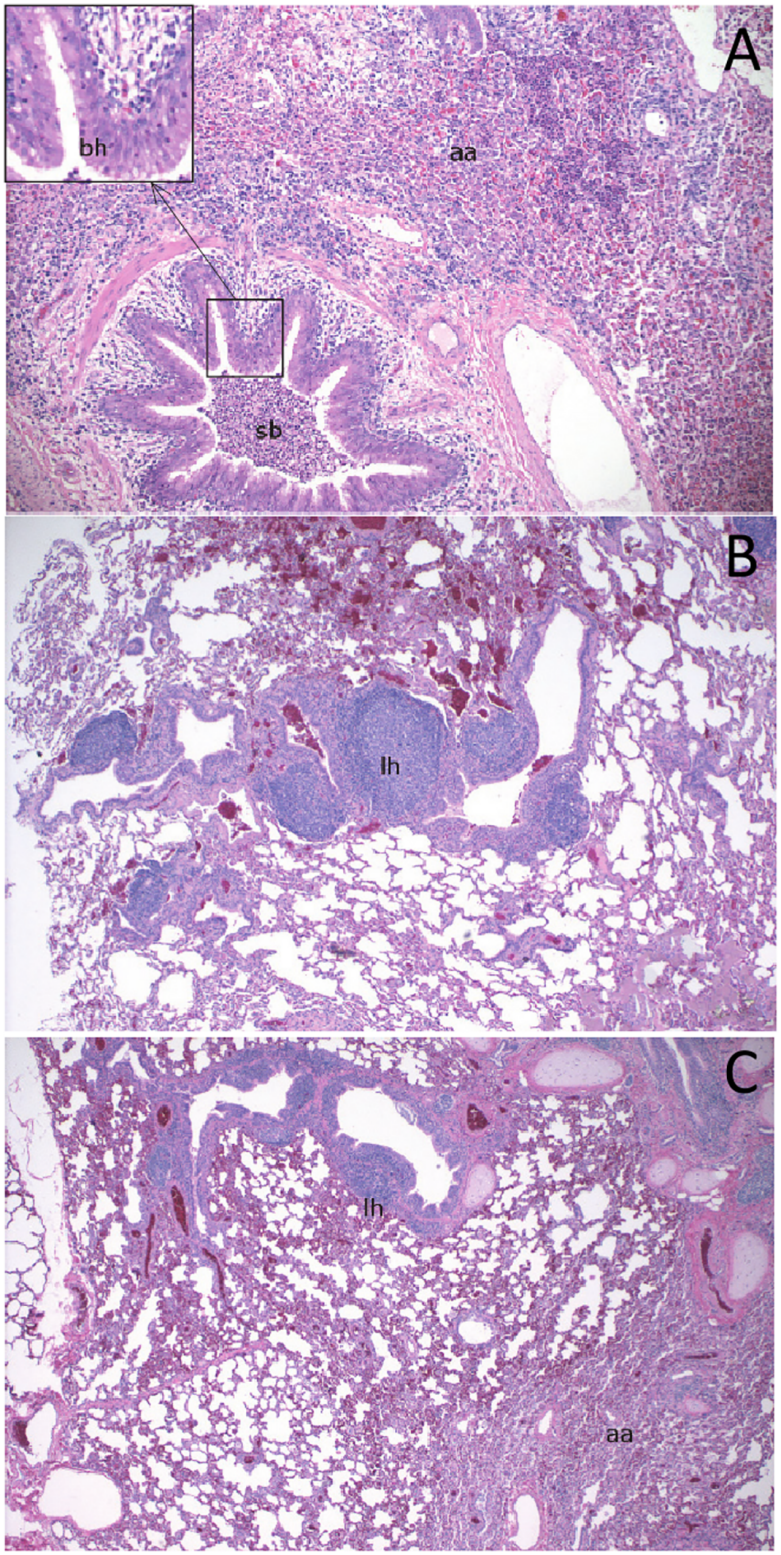
Representative histological lung lesions in experimental animals. Similar lesions were observed in all necropsied experimental animals. (A) BHS29 following experiment 1; (B) BHS28 following experiment 3, and (C) DG5 following experiment 3. aa = alveolar atalectasis; bh = bronchiolar hypertrophy; lh = lymphoid hyperplasia; sb = suppurative bronchiolitis.

Gross necropsy examination of the bighorn sheep in experiments 1 and 3 and in the domestic goats in experiment 3 revealed limited areas of lung consolidation in most but not all animals (Tables 1 and 3). These areas were localized to the ventral tips or edges of the middle and anterior lobes, and were darker red in color and firmer on palpation than the adjacent normal lung tissues. In addition, several animals had strong fibrous adhesions between these same lung regions and the pericardial or parietal pleura, presumably representing resolved pleuritis. All animals in the study had similar histopathologic lesions that varied in severity, consisting of inflammation centered around bronchi and bronchioles and extending to include adjacent alveoli (Fig 3). Inflammation was characterized by peribronchiolar and perivascular lymphoid hyperplasia with secondary suppurative bronchiolitis and alveolar atelectasis.

### Microbiology findings

Microbiology and serology confirmed *M*. *ovipneumoniae* infection in all BHS only after contact with colonized DG, which resulted in seroconversion to this bacterium in both BHS and previously unexposed DG. *M*. *ovipneumoniae* IGS genotypes detected at necrospy in bighorn sheep or domestic goats shared identical sequences with those detected in the source goats prior to the studies. In experiment 1, a single IGS genotype was detected in all domestic goats prior to and after the experiment, and in all bighorn sheep at necropsy after the experiment. In experiment 2, two different IGS genotypes were detected in the domestic goat inoculum source, one of which was subsequently detected in all commingled goats and bighorn sheep, while the other strain was detected in all the domestic goats but was not detected in any of the comingled bighorn sheep. Both the bighorn sheep and domestic goat groups were colonized in the oropharynx by diverse Pasteurellaceae prior to, during, and after these experiments, and some of these bacteria were also isolated from pneumonic lung tissues of the animals at necropsy (Tables 1 and 3). These included *lktA+ M*. *haemolytica*, sometimes considered the most lethal Pasteurellaceae [23, 28].

## Discussion

The results of this study both resemble yet substantially differ from those of previous studies of the impact of *M*. *ovipneumoniae* infection on initiation of pneumonia in bighorn sheep. Similar to previous studies exploring contacts with domestic sheep, comingling of *M*. *ovipneumoniae-*carrier domestic goats resulted in transmission of the pathogen to susceptible bighorn sheep, which then developed signs and lesions of respiratory disease [21]. However, contrary to previous studies that utilized domestic sheep carrying *M*. *ovipneumoniae*, the respiratory disease observed following experimental contact with domestic goats carrying *M*. *ovipneumoniae* was relatively mild, resulting in no fatalities. In addition, respiratory disease of comparable severity to that shown by the bighorn sheep also developed in naïve domestic goat hosts following infection, something that had not been reported in previous studies utilizing naïve domestic sheep. The lesions observed were consistent with those described in *Mycoplasma* spp. infections of sheep, goats and cattle, often called enzootic pneumonia. Enzootic pneumonia lesions are distinct from those caused by other bacterial agents of bronchopneumonia, such as *Pasteurella multocida* and *Mannheimia hemolytica*, which are characterized by widespread hemorrhage, necrosis and suppurative inflammation[13].

These contrasting results have interesting implications about the strain-specific virulence of *M*. *ovipneumoniae* and the specific roles of this and other bacterial pathogens in bighorn sheep respiratory disease. They also provide an area for additional research to identify management approaches for preventing new outbreaks of respiratory disease in bighorn sheep.

These results strengthen the previously documented links between exposure to *M*. *ovipneumoniae* and development of respiratory disease in bighorn sheep. All bighorn sheep exposed to goats carrying *M*. *ovipneumoniae* in experiments 1 and 3 developed signs and lesions of pneumonia, even when they had previously been exposed to the same goats in the absence of *M*. *ovipneumoniae*. All bighorn sheep kept in captivity under very similar conditions, but without contact with goats (experiment 1 pen 2) or in contact with *M*. *ovipneumoniae-*free goats (experiment 2, pen 1) did not develop signs of pneumonia. Pasteurellaceae bacteria carrying the gene encoding their principal virulence factor (*lktA* encoding leukotoxin) are another group of pathogens that have been hypothesized to play key roles in bighorn sheep pneumonia [23, 28], but both the bighorn sheep and the domestic goats used in these studies carried these pathogens prior to the experiments, and the presence of these pathogens were not clearly linked to the experimental outcomes or pathologic lesions.

Despite the consistent development of bighorn sheep pneumonia following contact with domestic goats carrying *M*. *ovipneumoniae*, the disease outcomes were more similar to those observed in the two previous experiments in which domestic goats were comingled with bighorn sheep and differed markedly from previous bighorn sheep-domestic sheep comingling experiments [4, 5, 8]. Unfortunately, the *M*. *ovipneumoniae* carriage status of the goats used in those previous experiments was not tested and no clinical observation data were reported. The cumulative 98% mortality reported for bighorn sheep comingled with domestic sheep (88 deaths among 90 bighorn sheep in 11 experiments as reviewed in [4]) was significantly higher than either the mortality rates of those two previous goat commingling experiments (2-tailed Fisher’s exact test, *P =* 0.0014 [5] and <0.0001 [8], respectively) or the two experiments reported here (each *P*<0.001). Therefore, while bighorn sheep comingled with *M*. *ovipneumoniae* carrier goats consistently developed respiratory disease and pneumonia, mortality was a rare outcome compared to that observed when bighorn sheep were comingling with domestic sheep carrying *M*. *ovipneumoniae*.

One hypothesis that may explain the differing severity of outcome of bighorn sheep infected with *M*. *ovipneumoniae* originating from domestic goats is that *M*. *ovipneumoniae* with differing virulence traits may be harbored by these two host species. Emerging evidence supports the existence of distinct clades of *M*. *ovipneumoniae* from these two host species [7, 14]. A naturally occurring bighorn sheep pneumonia outbreak linked to *M*. *ovipneumoniae* belonging to the domestic goat clade, described in [7], resulted in approximately 30% pneumonia-associated mortality in the adult bighorn sheep, similar to that reported in [5] and towards the lower end of the range of mortality rates reported in pneumonia outbreaks affecting wild bighorn sheep [3, 29–32].

Since impaired recruitment due to *M*. *ovipneumoniae-*associated annual lamb pneumonia mortality is recognized as a key population-limiting effect of pneumonia in bighorn sheep [11], we anticipated that the lambs born to the ewes comingled with domestic goats in these experiments would provide insights into the effects of goat-origin *M*. *ovipneumoniae* on lambs. In both pens, lambs experienced a similar range of diseases but no bronchopneumonia was observed in any lamb, and the deaths in pen 1 occurred prior to the age at which lamb pneumonia typically is seen. Therefore, the experiments were not informative about lamb pneumonia resulting from *M*. *ovipneumoniae* carried by domestic goats, although the infections experienced by the pen 1 lambs clearly documented pathogen transmission among domestic goats, bighorn ewes, and bighorn lambs. Further studies will be needed to determine the persistent effects of goat-origin *M*. *ovipneumoniae* on bighorn sheep lambs in the years following initial expsoure.

Conclusions and management implications: The severity of disease induced in bighorn sheep by the goat-origin *M*. *ovipneumoniae* strains in these experiments was relatively mild and transient. However, important questions remain: 1) Do other, more virulent strain-types exist within the goat *M*. *ovipneumoniae* clade? 2) Are goat-origin *M*. *ovipneumoniae* persistently carried by some bighorn sheep that recover from mild infections, as sheep-origin strains sometimes are? 3) If persistent carriers of goat-origin *M*. *ovipneumoniae* exist among bighorn ewes, are these strains subsequently transmitted to their lambs and do they induce lamb pneumonia? Answers to these questions will be relevant to the development of risk-appropriate management procedures to protect bighorn sheep from epidemic pneumonia.

## Supporting information

S1 Table Clinical scores of bighorn sheep and domestic goats. (XLSX)
