# Correction: Exposure of bighorn sheep to domestic goats colonized with *Mycoplasma ovipneumoniae* induces sub-lethal pneumonia

**DOI:** 10.1371/journal.pone.0192006

**Published:** 2018-01-24

**Authors:** Thomas E. Besser, E. Frances Cassirer, Kathleen A. Potter, William J. Foreyt

In response to queries raised after publication, the authors, the authors’ institution (Office of Research Assurance, Washington State University) and a member of PLOS ONE’s Editorial Board have reviewed the findings in this article, and as a consequence the authors provide an update to the Competing Interests statement and clarifications regarding the results:

The competing interests declaration is updated to acknowledge additional sources of funding received by the authors. This specific study was supported by funding competitively awarded by the Wild Sheep Foundation and by revenue from the WSU Rocky Crate Endowment for Wild Sheep Disease Research. Additional research funding for the authors’ bighorn sheep pneumonia-related research has been received from the US Department of Agriculture (including the Animal Plant Health Inspection Service and the US Forest Service), the US Geologic Survey, numerous chapters and affiliates of the Wild Sheep Foundation, and the WSU Fowler Emerging Infectious Diseases endowment.

Regarding the pneumonia diagnosis reported in the article, the authors re-assessed the primary data and solicited and received a second opinion from a veterinary pathologist at Washington Animal Disease Diagnostic Lab (WADDL) unassociated with the original project. Following this reassessment, the authors confirmed the descriptions and diagnoses as reported in the article, but they noted that the consulted pathologist advised, “some pathologists might describe the histopathologic lesions seen in the least severely affected animal as 'bronchiolitis' rather than 'pneumonia' due to the preponderance of that lesion in that animal”. In light of this, the final sentence of the “Necropsy findings” section (Results) should read, "Inflammation was characterized by peribronchiolar and perivascular lymphoid hyperplasia in all animals, with secondary suppurative bronchiolitis and alveolar atelectasis in the more severely affected animals."

There is inconsistency in reporting pleural adhesion lesions: the third sentence of the “Necropsy findings” paragraph notes that “several animals had strong fibrous adhesions between these same lung regions and the pericardial or parietal pleura…”. The word “several” is incorrect here, as this was observed only for two animals as was correctly specified in Tables 1 and 3.

These corrections do not affect any of the conclusions of the study.
